# Redefining Health: Implication for Value-Based Healthcare Reform

**DOI:** 10.7759/cureus.1067

**Published:** 2017-03-02

**Authors:** Ikhwanuliman Putera

**Affiliations:** 1 Faculty of Medicine, University of Indonesia

**Keywords:** health, outcomes, cost, quality of care, integrative medicine, value-based healthcare

## Abstract

Health definition consists of three domains namely, physical, mental, and social health that should be prioritized in delivering healthcare. The emergence of chronic diseases in aging populations has been a barrier to the realization of a healthier society. The value-based healthcare concept seems in line with the true health objective: increasing value. Value is created from health outcomes which matter to patients relative to the cost of achieving those outcomes. The health outcomes should include all domains of health in a full cycle of care. To implement value-based healthcare, transformations need to be done by both health providers and patients: establishing true health outcomes, strengthening primary care, building integrated health systems, implementing appropriate health payment schemes that promote value and reduce moral hazards, enabling health information technology, and creating a policy that fits well with a community.

## Introduction and background

The World Health Organization (WHO) defined health, in 1948, as “a state of complete physical, mental and social well-being and not merely the absence of disease or infirmity.” This definition of health has been receiving attention, as it seems obsolete in the 21st century, due to the emergence of chronic diseases in the aging populations. Nowadays, with the increasing number of risk factor exposures and the application of early screening methods, it is difficult to achieve “health” [1]. Chronic conditions currently account for three-quarter of the health expenditure worldwide [2]. This concept of health leads us to what extent we practice healthcare, including the adoption of the most suitable health delivery system and financing. The current definition also guides us on how we measure outcomes of medical interventions.

Based on the definition by the WHO, there are three aspects of health to consider: physical, mental and social health. Health in physical domain reflects the ability of individuals to maintain physiological homeostasis through changing conditions, or “allostasis”. Illness results from unsuccessful physiological coping mechanism during harmful circumstances. The mental domain states the sense of coherence to adapt and manage ourselves to improve subjective well-being. Finally, an individual's capacity to manage life is included in the domain of social health, where interaction with other living objects and environments take place [1]. All of these three domains of health determine how we measure health outcomes. Recent advances in health research bring us to the ever-changing new evidence-based medicine. The best evidence from meta-analysis or systematic reviews is based on well-conducted clinical trials. The evidence should direct us to not just one dimension of health; indeed, but all aspects should be considered in practice. A therapy may prolong survival rate but sacrifice functional status whereas another has slightly lower survival rate but improves functional status significantly. Physicians should consider all of these physical, mental, and social domains when deciding the best intervention.

The re-emerging “value-based healthcare” concept in recent years forces us to re-evaluate all aspects of our health practice, nowadays, to deliver and maintain the health of a population. The Economist Intelligence Unit defines value-based healthcare as “the creation and operation of a health system that explicitly prioritizes health outcomes which matter to patients relative to the cost of achieving this outcome” [3]. The concept of value-based healthcare questions the need of aggressive, preventive or curative interventions which cost a lot but have few outcomes, while being ineffective and inefficient medical practice. On the other hand, this urges us to not seek for services to lower cost while sacrificing outcomes. Modern healthcare also has four precepts: evidence-based, patients centered and inclusive of carers and the community, continuous and coordinated, and ethically sound and regulated [4]. This review aims to describe the current understanding of health practice to implement value-based healthcare.

## Review

### Health and outcomes are set for specific medical conditions which matter to patients

The ultimate goal of healthcare is to create a healthier society. Short-term goals such as improving access to healthcare and increasing profits have been distractions. As health is something that matters to patients, the goal of healthcare should be patient-centered and not provider-centered. The concept of value-based healthcare tries to increase health outcomes in an efficient way. Porter stated that quality assessment, somehow, does not reflect the actual “quality”. Instead, it is a measurement of a process that captures compliance with guidelines. The only true quality lies in the patients’ circle, that is, patients’ health outcomes [5].

The first important thing to consider in delivering value-based healthcare is defining the value that matters to patients. Physicians often think that they deliver healthcare well by increasing services, indicated by increasing visits. However, patients deserve good outcomes which are reflected not by more visits, procedures, or tests, but better health status and value. As value reflects outcomes per dollar spent, we should measure outcomes appropriately. We need a mechanism to report and evaluate risk-adjusted outcomes for each medical condition along with costs to achieve those outcomes. It should be noted that outcomes are not merely calculated from mortality and morbidity but also other multifactorial aspects significant to patients in a cycle of care, including complications, recovery time, and the need for further treatments. This outcomes report should also be stratified or risk-adjusted by observing the patients’ current condition. Outcomes are measured not for an individual service or intervention but for a full cycle of care [6]. 

Porter describes outcomes in three tiers. Tier 1 involves health status achieved, including mortality and functional status. Tier 2 involves the nature of care and recovery, including readmission and duration of return to normal daily activities. Meanwhile, Tier 3 relates to the sustainability of health. Providers should focus on all aspects of outcomes and not become easily satisfied with one outcome. For example, a longer five-year survival rate does not necessarily reflect the real health status; yet, we need to be concerned about the readmission, complication, and pain that exists, and the patient's ability to perform daily activities independently [5].

Assessing functional status could be challenging. Therefore, patient-reported outcomes (PROs) measurement has been introduced. The Patient-Reported Outcomes Measurement Information System (PROMIS) offers an efficient way to evaluate outcomes after medical treatments. This measurement includes three domains of health (physical, mental and social domain). The validity and reliability of this measurement have been evaluated in a previous study [7]. Using computerized adaptive testing (CAT), measurement of functional status after discharge could be individualized for every patient’s need in the future [8].

Currently, the standard sets of outcomes for specific medical conditions have been proposed by the International Consortium for Health Outcomes Measurement (ICHOM). ICHOM was founded in 2012 and has already been working on several medical problems by publishing comprehensive outcomes [9]. For example, ICHOM’s outcomes for coronary artery disease (CAD) and lung cancer have been published. In the CAD working group, they focused on short-term (hospitalization, 30 days post-discharge) and long-term (one-year and five-year survival) outcomes [10-11]. Besides clinically measured outcomes, they also included patients’ quality of life through several instruments (Seattle Angina Questionnaire, Rose Dyspnea Score, and Patient Health Questionnaire). In the lung cancer working group, the outcomes can be applied to non-small cell and small cell carcinoma patients. Survival, complications, and degree of health were measured by documenting patients’ quality of life and quality of end-of-life reported. Those outcomes were also adjusted for several risk factors determined by working groups.

### Strengthening the role of primary care in healthcare system

The earliest stage of primary care history in the early 20th century unintentionally defined primary care physicians or general practitioners (GP) as physicians who lacked further training. However, evidence shows that primary care is the cornerstone of a nation’s health and not specialty care. Regarding this evidence, many countries have tried to save their primary care with additional post-graduate training. In the 1960s, the United States added longer postgraduate training and established credentials for family practice specialty. The Institute of Medicine (IOM) then defines primary care as “the provision of integrated, accessible healthcare services by clinicians who are accountable for addressing a large majority of personal healthcare needs, developing a sustained partnership with patients, and practicing in the context of family and community.” There are the four main features of primary care: (1) first-contact access, (2) long-term person-focused care, (3) comprehensive care for most health needs, and (4) coordinated care [12].

Primary care mainly deals with preventive interventions. Despite fighting a disease and defining health as an output of medical interventions that restore health as described earlier in this paper, outcomes of primary care are measured based on its contribution to maintaining health in populations, especially those with risk factors. In line with our discussion about value-based healthcare, this topic also deserves attention. How we deliver effective yet preventive medicine with the lowest cost should become a priority. One of them could be increasing the number of primary care physicians as a higher primary care physicians' ratio appears to increase community health outcomes: lower infant mortality, low birth weight, and all causes of mortality [12]. Based on this evidence, strengthening primary care, with sustainable epidemiological research and development strategies that fit well with the community served, will result in the most effective way to maintain health. For example, if acute coronary syndrome (ACS) is known to be the leading cause of mortality and morbidity in a specific population, epidemiological research to find the most contributing risk factors for ACS in that population will be beneficial to plan the next strategy to reduce the risk. In this case, the most effective and preventive intervention with the lowest cost should be promoted. This cycle should always be carried out continuously.

As the first contact for medical needs, the role of primary care is important. Some countries have implemented a health system which encourages people to go to their primary care physicians before seeking care elsewhere. In this circumstance, we move our discussion from community health outcomes to disease-specific outcomes. Interestingly, health outcomes are found to be better where patients were referred by a primary care physician rather than self-referral to a specialist [12]. Roos (1979) suggested that children with recurrent tonsillitis or otitis media, who were referred by a primary care physician and needed surgery, were found to have fewer postoperative complications, fewer episodes of otitis media, and fewer postoperative respiratory problems [13]. The outcomes were not limited to the physical domain of health. Self-assessed health also indicated fewer people had less depression if they received adequate primary care services [12].

Critics often question the quality of clinical care by primary care physicians. If we measure outcomes with guideline adherence management for specific diseases, specialist care is better. For example, specialist care for managing asthma and helicobacter pylori infection is better compared to primary care. A study shows that this phenomenon can be reduced where GPs received additional training [12]. Again, we should not waste our time concluding about the quality of care by assessing performance. Instead, we should focus on a set of outcomes described earlier in this paper.

The current health system seems to underappreciate primary care practice. Improving value in healthcare system has gained much attention, but is not rewarded appropriately. Porter and colleagues suggested a better way to construct primary care in measuring outcomes and costs by way of acquired new skills, new ways of accessing patients, new payment schemes, and new approaches to integrate primary care with specialist care. Porter and colleagues pointed out that it is impractical to measure value in primary care due to heterogeneous patient groups in primary care settings, leaving primary care to deal with supply-driven participants in health. Transforming primary care to align with value-based health requires five essential efforts: (1) grouping patients based on similar needs not by diseases; (2) building a team focusing on care integration for each patient group, where specialists can also be involved in the team as part of care integration; (3) measuring value for each group; (4) implementing payment scheme suitable for value, in this case, Porter and colleagues support the use of bundled payment with a risk adjustment scheme in primary care while fee-for-service scheme is available for acute care, and; (5) integrating primary and specialist care. This concept requires collaborative work by primary care physicians instead of practicing alone [14].

### IPU vs IDS: value-based competition on healthcare delivery system

The concept of Porter and Teisberg has challenged the current healthcare system where providers compete to shift costs to others to increase bargaining power, to capture patients and restrict choices, and to reduce costs, but in the end not to create value. They proposed that a freestanding integrated practice unit (IPU) will boost value by competition in how healthcare is delivered to a medical treatment in a cycle of care [15]. In specific IPU, integrated medical care is provided through a multispecialist team who collaborate to achieve and compete for best possible outcomes with the lowest costs. Outcomes can be compared among IPUs covering a full cycle of care for specific medical problems.

IPU, as discussed in Porter’s paper, is defined as “a dedicated team made up of both clinical and nonclinical personnel who provide the full care cycle for the patient’s condition.” In a specific IPU, not only the disease but also all related conditions will be treated. The personnel are experts working together in trust and easily collaborating to reduce wasted time and resources. For example, in IPU dealing with lower back pain, a neurologist, an orthopedist and a rheumatologist work closely and collaboratively along with a physical therapist and a radiologist [5]. 

Enthoven and colleagues debated the idea of Porter and Teisberg. A full cycle of care described by Porter and Teisberg ideally starts from a disease or a specific medical condition. However, in practice, health and disease are not simple to define. This paper has already discussed the importance of primary care as patients nowadays have several chronic conditions which are probably beyond the scope of care of a specific IPU. In their review, Enthoven and colleagues questioned the efficiency of care if a patient has several medical problems. For example, if a patient has diabetes, heart disease, and depression at the same time, the question would be whether he/she should seek medical treatment in three different IPUs specializing in diabetes, heart disease, and psychiatric condition [16].

Therefore, integrated delivery system (IDS) to enhance value should be emphasized to challenge the IPU. Collaborative work with all participants, including physicians, pharmacists, and hospital play a central role to leverage value. Enthoven and colleagues explain that value can be added if orthopedic surgeons, for example, agree to study joint prostheses collaboratively, to select the best supplier, standardize surgeons, and train nurses for the best practice. Enthoven and colleagues revealed this integration in managing multiple conditions, especially chronic disease, enables IDS to have a higher value over freestanding IPU. Cost beyond a cycle of care, including diagnostic equipment, also can be shared in IDS [16].

Clinically integrated and collaborated care is essential for increasing value without overlooking competition. There is always space for patients to choose the best medical providers based on their reported value, especially outcomes, while primary care and specialist care stay integrated and collaborated. The patients’ capability to choose providers they want will ensure a good competition between providers. Patients should not be permanently assigned by a system to a PCP. Patients should be free to designate a PCP or change their PCP later if they want. This raises an idea of patient-centered care.

The term “patient-centered care” is preferable instead of “disease-oriented care”. Studies show that patient-centered care improves patients’ satisfaction, quality of care and health outcomes along with reducing costs and disparities. IOM defines patient-centered care as “respectful of and responsive to individual patient preferences, needs, and values, and ensuring that patient value guides all clinical decisions.” Patient-centered care encourages physicians to have a two-way relationship with patients by favoring what really matters to the patients, and ultimately supporting core elements of value-based health. Moreover, when treating patients with chronic and complex conditions, providers should not only focus on an individual physician-patient relationship, but also “communities of care.” A community of care reflects communication among physicians collaboratively and integratively [17].  

Then, which one is the best, IPU or IDS? The answer to this question is still unknown as evidence is still limited. Despite arguing which one is the best, we can gain as many positive advantages as we can from those theories. Both IPU and IDS will produce benefits if they are related to strong primary care: patients with early stage of diseases will have fewer comorbidities as preventive interventions for several medical problems have been delivered continuously with both approaches. Thus, the approaches can produce higher outcomes. With the increasing importance of primary care and the shifting population having multiple chronic diseases, integration of care should be prioritized. However, competition among providers to deliver as much value as they can and minimize services provided should also be given attention. Competition remains an important element to boost quality. Competition could also leverage value. Improved quality and process will decrease costs and enhance customer satisfaction. Published outcomes and reward-punishment mechanism can, possibly, leverage value even when there is no one-to-one freestanding IPU competition. Through competition, providers will consider implementing the most efficient way to deliver healthcare by reducing unnecessary costs.

However, creating an ideal competition in healthcare is not easy. The competition will fail to boost efficiency because health insurance companies pay for most of the healthcare costs [18]. Interestingly, a zero-sum-based situation also emerges as Porter and Teisberg describe how providers divide value instead of creating value [15]. The patients’ satisfaction is one component to drive competition. The patients’ perspective, despite being subjective, can be used to improve the quality of care [18]. Reporting value, including patients’ satisfaction and other outcomes, assures that both patients and providers have enough information and compare performance.

Enthoven and Tollen favor risk-adjustment prepayment schemes and the ability of patients to choose IDS. Prepayment scheme should reward providers for maintaining a healthy population and solving medical problems at the lowest costs. Despite emphasizing IPU, they favor IDS because it encourages ambulatory care for chronic conditions and reduces hospitalization. IDS provides coordinated care in a full cycle of care including home, inpatient and outpatient settings. Costs should be the sum of total costs in all settings, not only in one setting. Besides, IDS should also be conducted efficiently with the help of IT, especially electronic medical records [19].

### Implementation of appropriate healthcare payment scheme to improve value

Fee-for-service makes each component of healthcare delivery system both a cost and revenue center. This is because provided services would be reimbursed a la carte. Specialists and acute care service centers will benefit due to their capabilities to provide complex medical services. Meanwhile, under capitation payment, there is no revenue center anymore. Profits depend on capabilities of providers to get a financial contract from health maintenance organization, to attract patients, and to manage the expenditure of care under capitation payment rate [20]. However, there are possible pitfalls in these two payment methods. First, in the fee-for-service method, providers can leverage outcomes as much as possible without taking a look at costs. In the latter option of capitation payment, providers are expected to lower their expenditure by restricting services and possibly yielding lower outcomes. Hence, these two payment methods seem to be unmatched in delivering value-based healthcare.

Solving the above problems require a long process and will not be extensively discussed in this concise review. However, again, strengthening primary care is the focus in all payment schemes nowadays. For example, capitation payment needs adequate and high-quality primary care physicians to succeed. Capitation payment limits unexpected medical services which cost more. Financial risk through this mechanism is unbearable for single primary care physicians who practice alone and cannot find a way through integrated and collaborative care. Also, this system needs collaboration and integration between primary care and specialist physicians. Specialists should have an attachment to primary care, enabling a culture of cooperation and mutual education. Specialists should focus on primary care success in attracting patients and managing costs. Limiting unnecessary referrals from primary care and unnecessary services from specialists will benefit both and more importantly, enhance value. In their review, Robinson and Casalino stated that the situations can be achieved through a physician compensation mechanism based on overall groups, primary care specialists, and performance instead of bills charged by individual physicians [20].

In addition to fee-for-service, there is a pay-for-performance (P4P) scheme. P4P is defined in Eichler’s paper as “transfer of money or material goods conditional on taking a measurable action or achieving a predetermined performance target” [21]. Trisolini defines P4P as “an approach used to provide incentives to physicians and healthcare provider organizations to achieve improved performance by increasing quality of care or reducing costs.” P4P differs from fee-for-service as fee-for-service rewards physicians according to the high volume of services without measuring quality [22]. Under P4P, the carefully designed financial incentive for both providers and consumers has the potential to increase utilization and quality of healthcare, thus leveraging value. For a set of specific health targets, there is an additional financial incentive. Supply and demand of health services are constructing P4P discussion. In demand side, barriers in consumer demand must be overcome. The supply side of P4P talks about production function, transforming inputs toward health outcomes and providers’ behavior on delivering health. Meanwhile, demands are influenced by individual factors related to illness perception, appreciation toward preventive intervention, willingness and ability to seek appropriate healthcare. In this context, the social norm in community plays a role. There are also some other multiple determinants of demands, including costs, household income, and structure of community that drive attitude toward health. In P4P, it is important to include outcomes, not process, as the target for the incentive. P4P solutions, described by Eichler with many examples, may generate better and rapid results [21].

Financial incentives can take the form of rewards or penalization to motivate providers to move forward toward desired outcomes. Tsiachristas acknowledged that old fashioned payment schemes did not provide enough incentive to perform integrated care. As described earlier, capitation and fee for service payment methods have pitfalls in optimizing value. Tsiachristas described alternative payment methods to optimize integrated care. Different countries tried to incentivize many stakeholders in varied forms, including P4P, pay-for-coordination, and bundled payment [2]. The introduction of P4P in the UK greatly affected physicians, especially family practice, to improve the quality of care [23]. In many European countries, combining alternative payment methods was applied. Tsiachristas favored implementing blended payment methods, with risk-adjusted population–based global payment as a basic method, supported with combination of P4P and pay-for-coordination [2]. In theory, P4P can be cost-effective as long as value improvement is large enough. However, although it seems to be more suitable to implement in the context of value-based health, several pitfalls have been already explained by Eijkenaar and colleagues. Those include the possibility that providers will select healthy or more compliant patients, select aspects of care that are more incentivized and neglect others, crowd out providers’ intrinsic motivation, and ultimately, manipulate data on outcomes. Providers mainly deal with deprived area and low adherence patients who may receive less incentive due to factors outside the providers. Those inequalities may be reduced by giving rewards for performance improvement and risk-adjustment calculation to the patients [24].

In their review, Eijkenaar and colleagues acknowledged limitations of the current available data about the effectiveness of P4P. Further evidence is needed and the measured outcomes should be long-term outcomes [24]. Kindig, then, suggested that after the introduction of the P4P scheme, we can move further to incentivize non-medical determinant of health, or “pay-for-population health performance system.” He argues that the current P4P will not significantly increase the value because (1) it does not focus on population/community health outcomes and (2) population/community health outcomes are not merely the result of medical care [25].

### Information technology platform, geographic barrier, and readiness of community

The use of information technology (IT) in healthcare is relatively new than in other sectors. It is easy to hypothesize that IT adoption will be followed with improved value, yet there is “productivity paradox” in which technology may not improve performance although it is still debatable. Angst, et al. proposed the idea of integration of health information technology. In their paper, health information technology is defined as an information process that deals with the storage, retrieval, sharing and use of healthcare data for communication and decision making. Medical technology will turn into information technology when stored data in isolated medical technology become accessible as an information and communication network. For example, images from computed tomography (CT) scan will become integrated and interoperable within hospitals [26]. This idea supports our discussion about integrated healthcare that enhances value. Outcomes and cost data are essential in value-based health era. Patients will not need to perform rigorous and repeated diagnostic tests from one healthcare to another for getting an accurate diagnosis, thus reducing cost and time. The IT through the central electronic health record (EHR) also allows physicians to meet consultants or more expert physicians when needed. Disease registries are as important as enabling EHRs. Information technology with implementation of EHR would be an initial stepping stone to progress on integrated patient-focused care [3]. 

Despite the implementation of IT to integrate healthcare systems and conduct efficient healthcare, failure of healthcare delivery, especially in developing countries, still exists. Systems-level improvement of healthcare delivery does not appear in settings with inequity. People living in resource-poor settings face many obstacles to achieve health including poor nutrition, limited transportation, and social norms. In such resource-poor settings, Kim, et al. proposed a suitable framework to implement a value-based health system, namely: (1) care delivery value chain for medical conditions, (2) shared infrastructure, (3) align healthcare delivery with external context, and (4) design a system to optimize equitable economic and community development. The idea of care delivery value chain begins with prevention as an initial step of the cycle of care and ends with monitoring and managing the patients’ medical conditions. Interventions should not focus on one intervention as every chain takes part in improving value. Shared infrastructures could distribute and integrate healthcare delivery across sites. Infrastructures include clinics, district and referral hospitals, and community-based care [27].

As we discuss how to increase value in primary care, problems in developing countries with many rural areas keep emerging. Rural areas tend to have small primary care practices that are unable to reform toward value-based primary care. However, they can actively form a network to support their practice [14]. Community health workers can enhance value by bringing healthcare closer to patients. This is suitable in settings with scarce health personnel. Moreover, shared delivery also implies addressing multiple health problems that usually occur, for example, human immunodeficiency virus (HIV) / acquired immune deficiency syndrome (AIDS) and tuberculosis. These conditions affect outcomes to one another; value will increase if all related conditions are treated simultaneously. External factors also contribute significantly to health among people living in resource-poor settings. These include nutrition, geographical barriers, social disparities, etc. These problems need broad solutions which depend on specific population needs [27].

IDS can also solve physicians' shortage and geographical barriers in resource-poor settings. This is supported by the study of Chen, et al. who reported their success in delivering ophthalmic care in the Matsu archipelago, Taiwan, as it is a remote archipelago with limited access to healthcare. Using their system, ophthalmologists kept rotating to deliver eye care in a specific duration of time on a shift basis [28]. Thus, the author suggests expanding primary care in value-based healthcare by also utilizing IT and IDS to overcome obstacles in delivering the most efficient healthcare.

Studies indicate that there is a discrepancy between the poor and the rich to obtain good quality healthcare. Quality, as defined by the Institute of Medicine (IOM) (2001), is “the degree to which health service for individuals and populations increases the likelihood of desired outcomes and is consistent with professional knowledge.” Poor quality of healthcare can be the result of either structural elements of health, the process to transform structural inputs into health outcomes, or both. Interestingly, the Disease Control Priorities Project (DCPP) team identifies that developing countries are taking too much effort in structural elements, including building infrastructure, increasing the supply of doctors and providing health insurance while these only represent the intent to provide health services. Studies in five countries, including China, India, Philippines, El Salvador, and Mexico show interesting findings. Compliance with clinical guidelines about common health problems could occur in settings with lack of structural elements while non-compliance could also occur in settings with good structural elements. They used vignette cases and found variation in the quality of care among medical practitioners. In this case, outcomes and quality could be enhanced through interventions to increase physicians’ adherence to guidelines [21].

Recent research by The Economist Intelligence Unit (EIU) to evaluate 25 countries indicated that many countries are in their early stages of adopting value-based healthcare. Their evaluation was based on an assessment of a country that enabled context of policy and institution towards value-based healthcare, measurement of health outcomes, integrated and patient-focused care, and outcome-based payment. A country needs an institution which specifically sets and reviews guidelines, evaluates the impact of health intervention toward medical, economic, and ethical aspects, and provides sufficient funding for research to address health-related knowledge gaps to fully adopt value-based healthcare systems. These components favor richer countries; as EIU mentioned, richer countries with more portion of the gross domestic product (GDP) spent on healthcare, tend to align more with value-based healthcare system [3]. An initial study in the UK also reported the potential for improvement despite the introduction of incentive programs in deprived areas [23], and probably this will be promoted in developing countries where inequity and geographical challenges exist.

Another thing to consider in defining health is “value” embedded in the community. “Value” in this context refers to the needs, wishes, and expectation of individual patients based on their beliefs and cultural support. This “value”, sometimes, does not match the evidence-based medicine concept. As an example, some religions might not accept blood transfusion even in a life-threatening condition. Principles and concepts embedded in a group or society would be relatively stable and fixed. Implementing value-based healthcare should not direct us to overlook “value” in practice, as one of the core ideas of value-based health is patient-centered care [4].

Lastly, along with the discussion on well structured patient-centered care and associated health payment schemes, there is an issue of how to deal with what is termed as a moral hazard. As providers try to increase value, increase outcomes, and lower cost, there are several potential negative consequences that cause people to be less concerned about their health status and risk factors exposure. All that providers do in value-based healthcare will always depend on a patients’ attitude and behavior. This attitude and behavior might be the two big “risk factors” in diseases. Implementing the right health insurance with risk adjustment might be a solution to complement value-based healthcare delivery. Deber stated that there are several issues to consider when applying for insurance in healthcare: (1) patients could not expect to buy coverage for something they knew they would need, (2) financial fairness to subsidize those who act positively towards their health, and (3) cost control over patients’ actions [29].

## Conclusions

Although the WHO has released the definition of health, the perception toward health could vary across populations. We are now facing an aging population with chronic conditions and risk factors exposure. The precise definition of health will guide us as to how we run a healthcare system. The introduction of value-based healthcare term drives us to evaluate our business, to not only talk about price and cost but also emphasize outcomes. Outcomes are something that matter to patients, not physicians, and should cover all necessary things to patients in a full cycle of care. The concept of value-based healthcare must be realized with the implementation of an appropriate health payment scheme, inducing integrated and collaborated work by providers and the use of IT to deliver efficient healthcare. The concept of value-based healthcare aligns with patient-centered care that tries to humanize people rather than to look at health as a business commodity by providing a range of services. Outcomes do not depend solely on mortality, but also on the quality of life. This concept fits best with Hippocrates’s quote “To cure sometimes, to treat often, to comfort always.”

An illustration from Frist acknowledges several aspects of our discussion above. He tried to illustrate how we could conduct healthcare well nowadays. He described a patient who is compliant with his medication. He initially selected a primary care by comparing their online credentials, available performance rankings, and pricing from reliable information platforms. He also had his own electronic medical record which can be accessed by all health providers with his permission. If one day, he has myocardial infarction and this is out of his residence and with the usual medical provider, a nearby emergency department can access all the necessary medical data with his permission. The bill charged would then be paid by the insurer which is slightly higher than the competitors because of its recognized higher quality and performance [30]. Therefore, the author suggests expanding primary care in value-based healthcare by also utilizing IT and IDS to overcome obstacles in delivering the most efficient healthcare.
